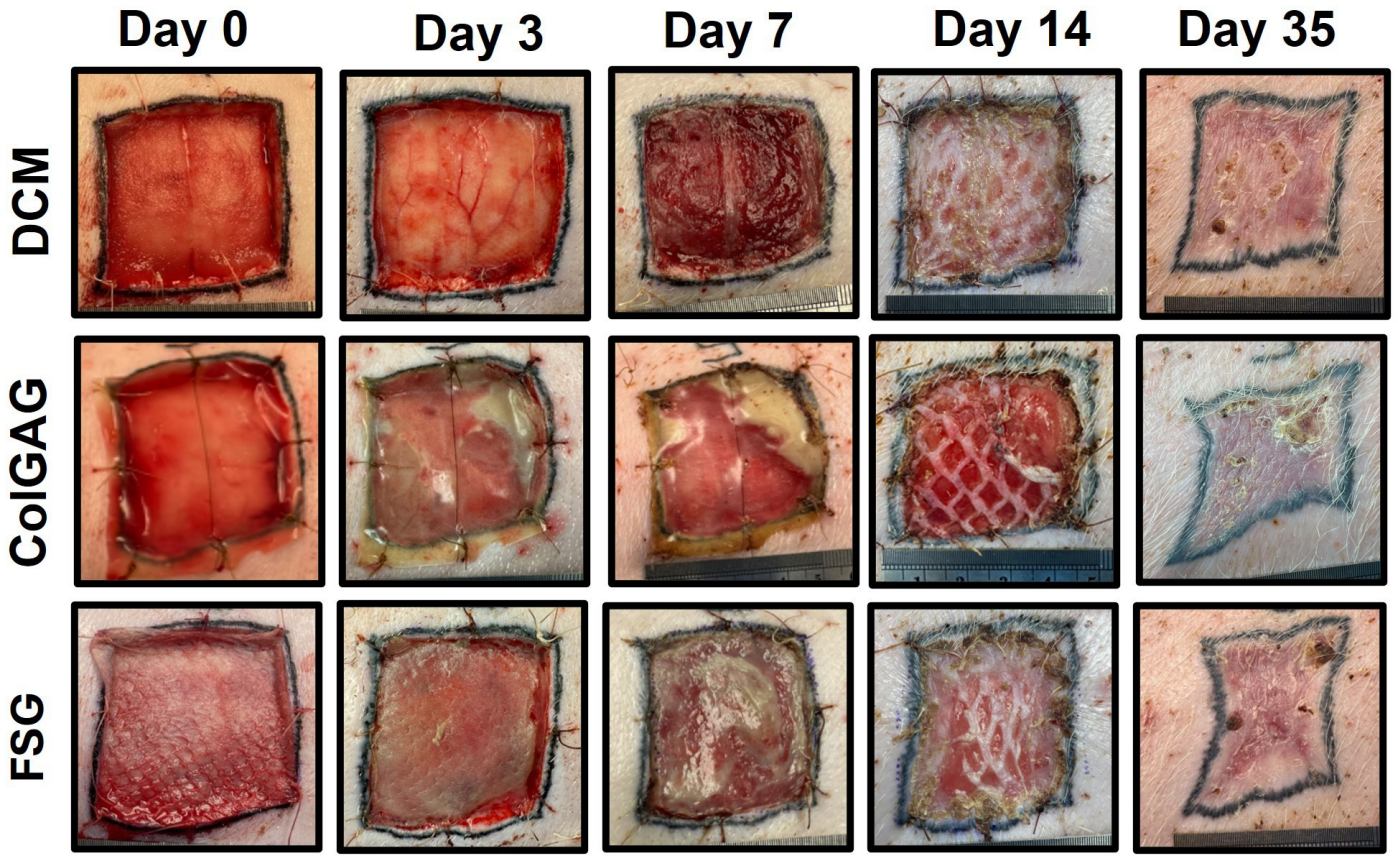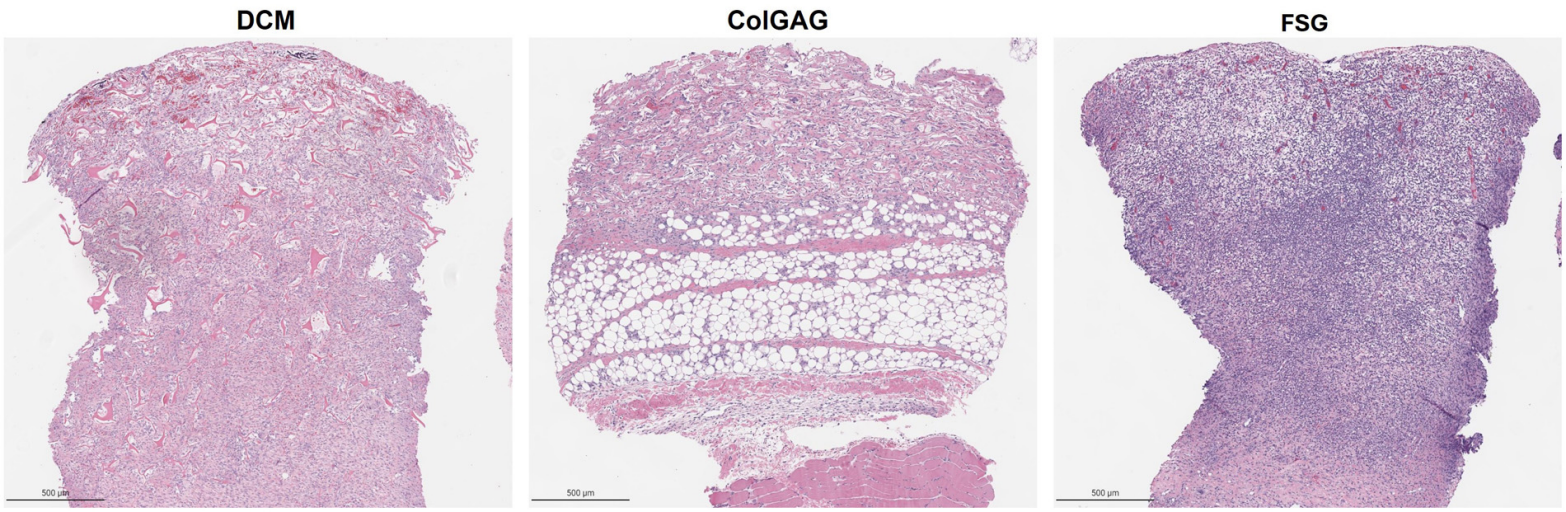# 64 Dermal Matrices Comparison: Evaluation of Integration and Autograft Take in Porcine Full-thickness Wounds

**DOI:** 10.1093/jbcr/iraf019.064

**Published:** 2025-04-01

**Authors:** Aleisha Chamberlain, Barbara Nsiah, Jayson Jay, Rachel Penny, Sohail Jahid, Ghaidaa Kashgari, Niraj Doshi, Katie Bush

**Affiliations:** AVITA Medical; AVITA Medical; AVITA Medical; AVITA Medical; AVITA Medical; AVITA Medical; AVITA Medical; AVITA Medical

## Abstract

**Introduction:**

Dermal matrices are commonly used in the management of full-thickness wounds. Various matrices exist having unique properties due to differences in raw materials and manufacturing techniques. These properties impact the body’s response to the implanted material leading to differences in time to tissue formation adequate to support an autograft.

A dermal collagen matrix (DCM) composed of dermal bovine collagen was developed to promote fast integration and decrease time to autografting (currently under FDA review). The purpose of this study was to evaluate response of DCM and impact on autograft take compared to other collagen based dermal matrices in a porcine full-thickness wound model.

**Methods:**

Full-thickness wounds (16 cm2) were created on the dorsum of Yorkshire pigs and treated with DCM (n=8), bovine tendon collagen (ColGAG) (n=10) or fish skin graft (FSG) (n=5) dermal matrices. At day 7, wounds were biopsied, debrided to obtain punctate bleeding, and a 3:1 meshed split-thickness autograft combined with autologous skin cell suspension was used for closure. Wounds were assessed and scored for matrix integration, infection, tissue fill, autograft take, and re-epithelialization. Planimetry was used to track size. Histopathological evaluation was performed.

**Results:**

Over the first 7 days all matrices had adherence to the wound beds; however, tissue integration and infection were found to differ (Figure 1left). The DCM incorporated into the wound developing into a red vascularized tissue with no infection. ColGAG had good integration, but infection was present in 70% of the wounds. FSG had limited integration (20% had ≥75% integration), but no infection was present. Histological evaluation at Day 7 indicates presence of a collagen fiber network populated with cells and blood vessels for DCM and ColGAG (Figure 2). Cells were present, but no three-dimensional matrix was detected with FSG.

Autograft take was more consistent for DCM compared to ColGAG and FSG with means of 96.88±7.04% compared to 85.00±34.10% and 68.00±27.97%, respectively (Figure 1right). Coefficient of variation was found to be 7.3% for DCM compared to approximately 40% for ColGAG and FSG. The percentage of wounds achieving ≥75% re-epithelialization at this time point was 100% for DCM, 60% for ColGAG, and 40% for FSG. No statistical differences were observed for contraction between dermal matrices at 35 days.

**Conclusions:**

DCM provides a microenvironment for healthy, infection-free formation of a well-vascularized tissue supportive of early autografting in a porcine full-thickness wound model.

**Applicability of Research to Practice:**

Following FDA clearance, DCM has potential to be a treatment option for full-thickness wounds which may enable grafting at earlier timepoints and minimize incidence of infection compared to other dermal biological dermal matrices.

**Funding for the Study:**

AVITA Medical